# Transrectal dual-plane ultrasound with contrast enhancement in primary vaginal fibroma diagnosis: A case report

**DOI:** 10.1097/MD.0000000000045820

**Published:** 2025-11-07

**Authors:** Xiaoting Chen, Jiang Zhu

**Affiliations:** a Department of Ultrasound, Women’s Hospital, Zhejiang University School of Medicine, Hangzhou, China.

**Keywords:** fibroma, transrectal biplane ultrasound, Vaginal tumor

## Abstract

**Rationale:**

Superficial vaginal fibroblastoma (SCVM), a rare benign mesenchymal tumor, is notoriously challenging to diagnose due to its asymptomatic presentation and location. Typically seen on ultrasound as a well-defined, homogeneous, medium-echogenic mass with abundant vascularity, it is often missed by conventional transvaginal ultrasound (TVUS) with end-fire probes, which inadequately assess the vaginal canal. To address this, we employed a novel integrated approach using transrectal biplane ultrasound (TRBU) and contrast-enhanced ultrasound (CEUS). This strategy significantly advances the early and accurate diagnosis of vaginal fibromas.

**Patient concerns:**

A 54-year-old female with no obvious symptoms presented for evaluation of a vaginal wall mass detected during a routine physical examination.

**Diagnosis:**

TRBU revealed a 30 mm-diameter hypoechoic mass on the left vaginal wall. CEUS demonstrated a hyperperfused mass with a capsular appearance, which was presumed to be benign. The patient’s HPV test was negative, and laboratory investigations showed tumor markers within normal ranges.

**Interventions:**

The patient underwent vaginal lesion resection, which was confirmed histopathologically as a vaginal soft tissue fibrous tumor. Following pathological departmental discussion, the lesion was favored to be a vaginal superficial myofibroblastoma.

**Outcomes:**

The patient had an uneventful postoperative recovery, and telephone follow-up confirmed no evidence of recurrence to date.

**Lessons:**

This case highlights the essential role of combining TRBU and CEUS in diagnosing indeterminate vaginal masses. The high-resolution imaging of TRBU, complemented by the real-time quantitative microvascular perfusion data from CEUS, allows for a thorough and nuanced evaluation. This combined approach delivers critical insights for accurate diagnosis and subsequent surgical decision-making.

## 1. Introduction

Vaginal masses are relatively rare in clinical practice, including benign lesions (such as fibroepithelial polyps (FPs), leiomyomas, vaginal cysts), malignant tumors (such as squamous cell carcinoma, adenocarcinoma, sarcoma), and special types of tumors (such as SCVM).^[1]^ Imaging plays a crucial role in detecting, localizing, characterizing, and staging these lesions. TRBU provides high-resolution images that visualize the layers of the vaginal wall, enabling assessment of vaginal tumor size, margins, invasion depth, and relationships with adjacent structures (e.g., rectum, bladder). Intravenous CEUS utilizes microbubble contrast agents to dynamically visualize tumor microvascular perfusion and assess hemodynamic features.^[2–4]^ Magnetic resonance imaging(MRI) can provide multi-parametric diagnostic information for further evaluating the staging of vaginal tumors and planning surgical/radiotherapy interventions. For morphological assessment and blood flow analysis of vaginal masses, TRBU and CEUS are often the preferred diagnostic modalities.

SCVM is a rare benign mesenchymal tumor that primarily occurs in the female lower genital tract, including the vagina, cervix, and vulva. In 2001, Laskin first reported SCVM, identifying it as an independent entity distinct from other tumors of the female reproductive system.^[5]^ We report a case of a 54-year-old female who presented with an asymptomatic vaginal wall mass discovered during a routine physical examination.

## 2. Case description

### 2.1. Patient characteristics and clinical presentation

A 54-year-old female patient was admitted to the hospital for diagnosis and treatment after a vaginal wall mass was detected during a physical examination. She had been diagnosed with breast cancer 8 years prior and underwent a left radical mastectomy. Pathological examination confirmed invasive breast cancer, and she received postoperative tamoxifen therapy (20 mg/day, for 4.5 years). Gynecological examination revealed a pedunculated mass about 3 cm in diameter, seen near the cervix on the left lateral wall of the vagina, with relatively good mobility and no tenderness.

### 2.2. Imaging findings

**Figure 1.**
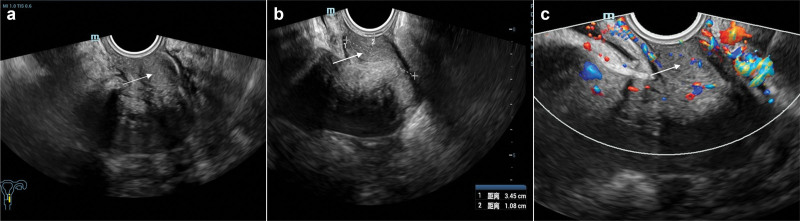
TVUS images. (A) A mass on the left vaginal wall (white arrow); (B) the mass measures 3.5 × 1.1 cm, with clear boundaries and a relatively regular shape; (C) color Doppler shows blood flow signals within the mass. TVUS = transvaginal ultrasound.

TVUS was performed(Mindray Resona-8EXP system with a 3–9 MHz probe), revealing a 3.5 × 1.1 cm mass on the left vaginal wall with intralesional blood flow signals (Fig. 1). Due to inadequate visualization of the mass’s relationship with surrounding tissues, TRBU was subsequently performed(Mindray Resona-8EXP system, convex array probe: 3–9 MHz; linear array probe: 4–13 MHz). By rotating the probe to orient the linear array transducer toward the left vaginal wall, the following findings were observed: the left posterior vaginal wall mass exhibited clear boundaries and smooth margins, with distinct separation from the rectum and anterior vaginal wall. Abundant blood flow signals were detected within the mass, showing a vascular resistance index of 0.48, regular vascular architecture, relatively uniform distribution, and intact basement membrane.

**Figure 2.**
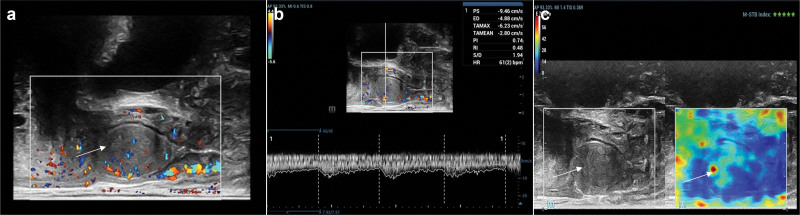
TRBU with elastography. The white arrow indicates the vaginal mass. (A and B) Abundant blood flow signals were detected within the mass, with a vascular RI of 0.48, regular vascular structure, and relatively uniform distribution; (C) elastography showed that the central region of the mass exhibited a local red area in terms of tissue hardness distribution, while the peripheral area showed a gradual decrease in hardness, transitioning from yellow to green. RI = resistance index, TRBU = transrectal biplane ultrasonography.

**Figure 3.**
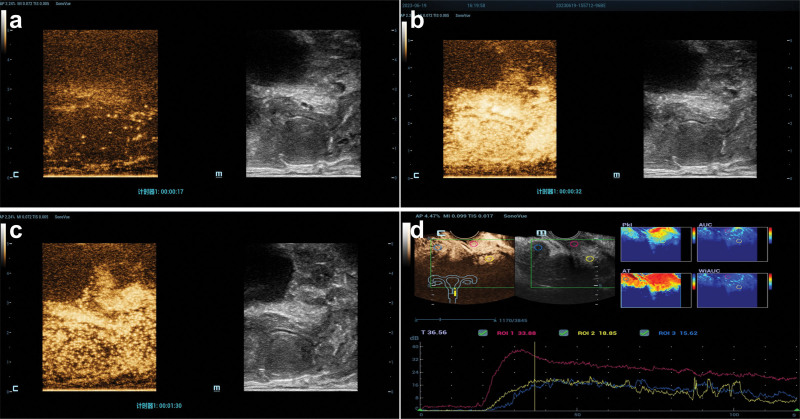
Transvenous contrast-enhanced ultrasound images. (A-C) After contrast agent injection, the mass began to show “centripetal” filling at approximately the 17th second, reached the peak at approximately 32 s, and exhibited slow, regular fading in the late phase with a capsular appearance; (D) perfusion data at the mass: time to peak: 32.4 s, peak intensity: 37.6 dB, AUC: 2654 dB; perfusion data at the cervix: time to peak: 54.4 s, peak intensity: 19.4 dB, AUC: 1267 dB; perfusion data of the uterine myometrium: time to peak: 55.3 s, peak intensity: 19.0 dB, AUC: 1294 dB.

Elastography revealed a localized red area in the central region of the mass with a gradual decrease in tissue stiffness toward the periphery, transitioning from yellow to green, resembling a “hard core with soft edges” pattern (Fig. 2). Subsequently, 2.4 mL of the ultrasound contrast agent SonoVue (a microbubble contrast agent manufactured by Bracco, Italy) was injected at a mechanical index of 0.078, followed by continuous observation for 120 seconds. The mass exhibited uniform hyperperfusion with a capsular appearance during the late-phase wash-out. Perfusion parameters of the mass: Time to peak: 32.4 seconds, Peak intensity: 37.6 dB, area under the curve: 2654 dB; Perfusion parameters of cervix: Time to peak: 54.4 seconds, Peak intensity: 19.4 dB, Auc: 1267 dB; Perfusion parameters of uterine myometrium: Time to peak: 55.3 seconds, Peak intensity: 19.0 dB, Auc: 1294 dB (Fig. 3). The radiologist interpreted these findings as consistent with a benign vaginal lesion.

### 2.3. Surgical and pathological outcomes

**Figure 4.**
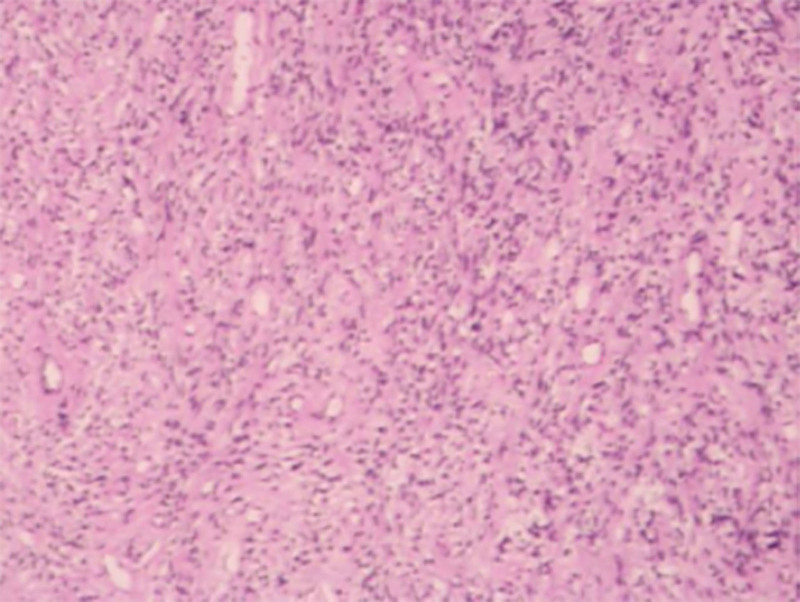
Postoperative pathology confirmed the lesion as a fibroma.

The patient underwent vaginal lesion electroresection, with an intraoperative blood loss of 5 mL and an operative duration of 65 minutes. Intraoperatively, an ovoid mass was identified on the left vaginal wall near the cervix, measuring approximately 3 × 2 cm, with a tough texture, white color, and a pedunculated base. The pedunculated mass on the left anterior vaginal wall was exposed and secured, and the pedicle of the vaginal mass was completely resected using electrocoagulation with an electrosurgical knife. Gross observation: The lesion appeared round-like, measuring approximately 3 × 2 cm, with a firm texture, white color, and whorled cross-section. Postoperative pathology revealed vaginal soft tissue fibroma (Fig. 4). Immunohistochemical staining showed: CD34 (-), SMA (+), Ki-67low (<1%), Bcl-2 (+). The patient is currently recovering well. The timeline of diagnosis and treatment is as follows (Table 1). This study was approved by the Ethics Committee of Women’s Hospital, School of Medicine, Zhejiang University (Ethics No: IRB-20240268-R).

**Table 1:** The timeline of diagnosis and treatment.

Date	Event	Key details
May 28, 2023	Initial detection	Asymptomatic vaginal mass (3 cm), breast cancer history, and long-term tamoxifen use.
June 19, 2023	TRBU-CEUS imaging	Gray-scale US: 3.5 × 1.1 cm hypoechoic nodule, well-defined, capsular sign.
		Elastography: soft
		CEUS: Hyperperfusion, TTP 14 s, peak 20 s, late-phase capsular enhancement.
June 20, 2023	Surgical resection	Transvaginal excision: Pedunculated mass (3 × 2 cm), whitish, whorled pattern. Blood loss: 5 ml; duration: 65 min.
June 28, 2023	Pathology confirmation	Fibroma diagnosis: Spindle cells, SMA (+), Bcl-2 (+), CD34(-), Ki-67 low (<1%).
June 29, 2023	Discharge	No complications; wound healing is normal.
January 10, 2025	Follow-up	No recurrence via transvaginal US; patient asymptomatic.

CEUS = contrast-enhanced ultrasound, SMA = smooth muscle actin, TRBU = transrectal biplane ultrasonography.

## 3. Discussion

SCVM is a rare benign mesenchymal tumor that typically arises within the submucosal stroma of the vagina and uterine cervix in females and is highly prone to misdiagnosis. SCVM predominantly affects perimenopausal and postmenopausal women, with the primary site being the vagina, followed by the uterine cervix; it can also occur in the vulva.^[5–7]^ Most patients present with a solitary mass, while a minority exhibit multiple nodules. SCVM has a wide age range at onset and varying disease durations, with the vast majority of patients developing the tumor during perimenopause or postmenopause. The primary clinical symptom is often a painless mass, although vaginal bleeding or discharge may also be observed. The incidence of SCVM is low, and its pathogenesis remains unclear. Some patients with SCVM have a history of tamoxifen therapy or hormone replacement therapy following breast cancer surgery, and this case conforms to this pattern.^[8]^ Liu et al confirmed that the development of SCVM is not associated with HPV, Epstein–Barr virus, herpes simplex virus, cytomegalovirus (CMV), or human herpes virus 8 (HHV-8).^[5]^

The clinical manifestations, signs, and auxiliary examinations of SCVM lack specificity, making preoperative diagnosis relatively challenging. Therefore, postoperative pathological examination is often required to further confirm the diagnosis. Ultrasonography serves as the most important imaging modality in preoperative diagnosis and management, as it can provide detailed information regarding the tumor’s morphology, boundaries, internal echogenicity, and blood flow characteristics, thereby holding significant value for preoperative evaluation and diagnosis.^[9]^ In this case, the mass was observed on ultrasound to have clear boundaries, a regular shape, and uniform internal echoes. Color Doppler ultrasound demonstrated moderately abundant blood flow signals with a resistance index of 0.48, consistent with previously reported ultrasound findings for SCVM.^[10]^ TVUS exhibits excellent performance in evaluating gynecological superficial lesions, with a sensitivity of 68% for detecting myometrial invasion in low-grade endometrial cancer, comparable to MRI, while costing only one-fifth as much as MRI. In terms of spatial resolution and imaging characteristics, TRUS utilizes a convex + linear biplane design, achieving a near-field spatial resolution of 0.5 to 1 mm. Although MRI offers significant advantages for soft tissue examination, it is expensive, reduces patient compliance, and complicates repeated examinations.^[11]^ Transabdominal and transvaginal ultrasound are commonly used in gynecological examinations, but both have limitations when evaluating vaginal lesions. Due to the vagina’s relatively deep anatomical location and the lower frequency of transabdominal ultrasound probes – resulting in reduced resolution – clear visualization of vaginal structures cannot be achieved. TVUS employs a convex endocavitary probe that emits sound waves from its tip toward the patient’s cephalic region, focusing primarily on uterine and adnexal assessment rather than comprehensive vaginal evaluation. Additionally, in patients with vaginal stenosis caused by lesions, probe insertion becomes impossible, precluding examination entirely. In contrast, TRBU can clearly delineate the vaginal layers and the relationship between masses and these layers, offering a new and effective approach for evaluating vaginal lesions. In this case, intravenous CEUS revealed regular vascular architecture and uniform enhancement pattern within the mass, characterized by “centripetal filling” and high perfusion. A clear demarcation was observed between the mass and surrounding tissues without evidence of invasion or compression, consistent with benign features.^[12]^ Analysis of CEUS perfusion parameters demonstrated a significantly shorter TTP in the mass compared to normal cervical tissue, indicating much faster blood flow velocity and more active local vascular hemodynamics. The pulsatility index within the mass was approximately 1.9 times that of the cervix, suggesting higher vascular density and more abundant blood perfusion. Additionally, the area under the curve of the mass was approximately 2.1 times that of the cervix, reflecting a significantly higher total contrast agent accumulation and overall greater blood perfusion volume compared to normal cervical tissue. These imaging features indirectly indicate that the mass has not infiltrated the surrounding tissues, and no other abnormal echoes were detected in the vaginal wall adjacent to the mass. These findings provide important guidance for surgical planning, leading clinicians to ultimately decide on transvaginal resection of the mass directly from its pedicle. Postoperative microscopic examination revealed a well-defined mass with a clear demarcation from the surrounding normal vaginal wall tissue and no infiltrative growth. The combined SMA (+) and Bcl-2 (+) phenotype strongly supports the diagnosis of a benign tumor of smooth muscle or myofibroblastic origin, consistent with previous literature reports.^[13]^

Many genital tract mesenchymal tumors exhibit significant overlap in their imaging and histological features, which complicates the differentiation between benign lesions and more aggressive subtypes.^[14]^ These tumors include aggressive angiomyxoma (AAM), angiomyofibroblastoma (AMF), FP, cellular angiofibroma (CAF), and SCVM described in this case.^[10,15]^ AMF predominantly occurs in the vulva/perineum of reproductive-aged women and is rarely found in the cervix/vagina. On ultrasound, it presents as a well-defined, round or oval hypoechoic mass, sometimes with partial encapsulation. Elastography shows that it is stiffer than the surrounding tissues, and blood flow signals are distributed either peripherally or internally.^[12,16]^ CAF is more commonly found in the vulvar/inguinal region of elderly women. Ultrasonography reveals a well-defined hypoechoic mass with heterogeneous internal echoes, and punctate, strip-like, or abundant blood flow signals are visible. Pathologically, it is characterized by thick-walled blood vessels and fascicular arrangement of spindle cells, surrounded by fat.^[17,18]^ AAM is a tumor with malignant potential, boasting a recurrence rate as high as 50%, and it typically arises in the perineum/deep pelvic region. Its typical ultrasonic features include ill-defined, layered, or whorled moderate to high echoes with infiltrative growth. Elastography demonstrates soft texture, CEUS shows high perfusion, and blood vessels exhibit a disordered course.^[19,20]^ FP is frequently observed in young women. Ultrasonographically, it presents as a well-defined hypoechoic mass, which is hard in texture due to its rich fibrous components, with scarce blood supply and low mobility.^[21]^ The ultrasonic differential characteristics are presented in Table 2.

**Table 2:** Ultrasonic differentiation features of five mesenchymal tumors in the vagina (SCVM, AMF, CAF, AAM, FP).

Ultrasound features	SCVM	AMF	CAF	AAM	FP
Internal echo	Moderate echo	Mainly hypoechoic with uneven intensity	Hypoechoic with uneven internal echoes	Medium-high echoes, layered/whirlpool-like	Hypoechoic
Boundary feature	Clear	Clear	Clear	Not distinct, showing invasive	Clear
Morphological regularity	Oval or round	Round and regular	Circular and regular	Irregular, lobulated	Polypoid and pedunculated
Blood flow signal	Rich with RI 0.47–0.52	Abundant and strip-distributed	Dot-like and strip-like with moderately abundant echoes	Rich and chaotic	Scant or none
Elastic hardness	Softer than the surrounding tissues	Higher than the surrounding tissues	No specific description	Softer than the surrounding tissues	Firm (rich in fiber)
Special ultrasound manifestation	A mucus isolation band between it and the mucosa	Deformed and displaced upon compression	Uncertain posterior echo attenuation/enhancement	Layered/whirlpool-like structure	Cluster of strong echo points (if with fat)
CEUS	complete and uniform isoechogenic enhancement and delayed fading	Slow in and slow out with uniform enhancement	Moderately heterogeneous enhancement	Rapid wash-out with a chaotic vascular network	No enhancement or mild marginal enhancement

AAM = aggressive angiomyxoma, AMF = angiomyofibroblastoma, CAF = cellular angiofibroma, CEUS = contrast-enhanced ultrasound, FP = fibroepithelial polyp, SCVM = superficial vaginal fibroblastoma.

In gynecological practice, CEUS demonstrates significant safety, cost-effectiveness, and clinical accessibility. The microbubble contrast agents employed (e.g.sulfur hexafluoride) are non-nephrotoxic, with a severe allergic reaction rate below 0.0001%, and do not cross the placental barrier, ensuring high safety for pregnant patients. Regarding cost, CEUS examinations cost only 1/3 to 1/5 of contrast-enhanced CT/MRI, with short procedure times (average 35 minutes), reducing the need for additional imaging tests by 50%. For accessibility, CEUS can be implemented through upgrades to conventional ultrasound equipment and performed on an outpatient basis without hospitalization requirements.^[22,23]^

**Figure 5.**
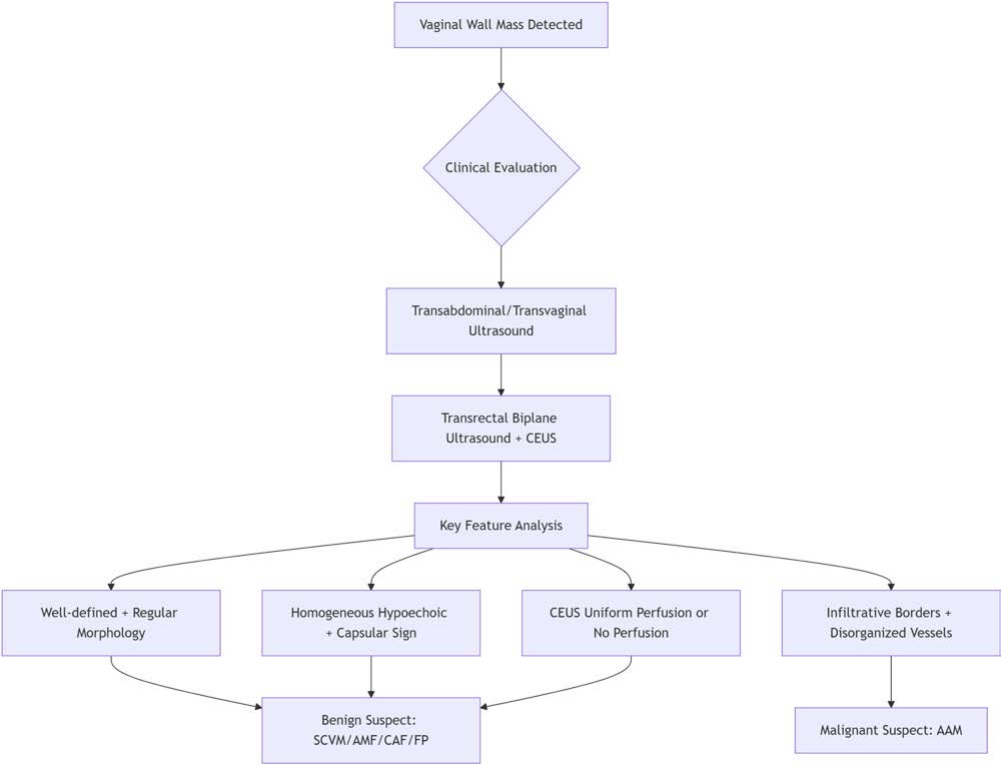
Diagnostic flowchart for vaginal masses.

As an inexpensive, convenient, and highly safe diagnostic tool, ultrasound utilizes high-resolution imaging via transrectal biplanar probes combined with intravenous CEUS technology to clearly visualize lesion location, size, echo characteristics, blood flow information, and relationships with surrounding tissues. This provides clinicians with high-precision diagnostic evidence and plays a crucial guiding role in surgical planning, thereby aiding in the differentiation of tumor types. The diagnostic flowchart is presented below (Fig. 5). This information is crucial for preoperative tumor characterization and surgical planning. However, a definitive diagnosis still relies on the results of pathological examination and immunohistochemical analysis. For the treatment of SCVM, surgical resection is the primary approach, with most patients experiencing favorable postoperative outcomes and a relatively low risk of tumor recurrence or spread.^[8]^

## 4. Limitation

In this study, standardized marginal assessment and histological subtyping were not routinely performed. This reflects the practical constraints and evolving pathological protocols in clinical practice at the time. Future prospective studies will follow the CAP cancer protocol reporting criteria to ensure consistency and comprehensive data collection. Furthermore, the generalizability of our conclusions is constrained by the small sample size (n = 1), which precludes statistical analysis and broad applicability. Therefore, validation through larger, multi-center prospective cohorts is crucial to confirm the diagnostic and clinical utility of the methods described in this paper.

## Author contributions

**Conceptualization:** Xiaoting Chen, Jiang Zhu.

**Investigation:** Xiaoting Chen.

**Methodology:** Xiaoting Chen.

**Resources:** Xiaoting Chen, Jiang Zhu.

**Supervision:** Xiaoting Chen, Jiang Zhu.

**Writing – original draft:** Xiaoting Chen.

**Writing – review & editing:** Xiaoting Chen, Jiang Zhu.
